# Early reduction in total cholesterol to high-density lipoprotein cholesterol ratio predicts hydroxychloroquine efficacy in treating IgA nephropathy

**DOI:** 10.1080/0886022X.2024.2397046

**Published:** 2024-08-30

**Authors:** Yaotong Shi, Ting Yang, Yuan Feng, Nan Li, Qiuyuan Shao, Chunming Jiang, Jing Liu

**Affiliations:** a Department of Nephrology, Nanjing Drum Tower Hospital, Medical School, Jiangsu University, Zhenjiang, Jingkou District, China; b Department of Nephrology, Nanjing Drum Tower Hospital, Affiliated Hospital of Medical School, Nanjing University, Nanjing, China; c Department of Pharmacy, Nanjing Drum Tower Hospital, Affiliated Hospital of Medical School, Nanjing University, Nanjing, China

**Keywords:** TC/HDL ratio, IgA nephropathy, hydroxychloroquine, prognostic value

## Abstract

**Background:**

Hydroxychloroquine (HCQ) effectively improves lipid levels in patients with autoimmune diseases. This study aimed to examine the effect of HCQ on lipid profiles in patients with immunoglobulin A (IgA) nephropathy (IgAN) and determine whether alterations in lipid profiles can predict the efficacy of HCQ.

**Methods:**

This study retrospectively analyzed 77 patients, and the total cholesterol to high-density lipoprotein cholesterol (TC/HDL-C) decline rate after 3 months of HCQ treatment was selected as a predictor based on receiver operating curve analysis. Patients were then divided into low and high TC/HDL-C decline rate groups based on the optimal cutoff value. The Cox proportional hazard model and Kaplan–Meier curve were used to evaluate the value of the TC/HDL-C decline rate in predicting the efficacy of HCQ in patients with IgAN.

**Results:**

Patients in the high TC/HDL-C decline rate group with ≥50% decrease in proteinuria from baseline experienced a significant improvement during the follow-up. Kaplan–Meier analysis revealed that a high TC/HDL-C decline rate was strongly associated with a higher proteinuria reduction rate in patients with IgAN. Furthermore, multivariate Cox analysis indicated that a higher reduction in the TC/HDL-C ratio (hazard ratio: 2.314; 95% confidence interval: 1.234–4.340; *p* = 0.009) was an independent predictive indicator for achieving ≥50% reduction in proteinuria with HCQ therapy in IgAN.

**Conclusion:**

HCQ effectively improves lipid profiles in patients with IgAN, and an early decrease in the TC/HDL-C ratio serves as a predictor of better outcomes in patients treated with HCQ.

## Introduction

Immunoglobulin A (IgA) nephropathy (IgAN), the most prevalent type of primary glomerulonephritis worldwide, is associated with a higher risk of renal failure [1]. Proteinuria is an important hallmark of renal damage in IgAN, and the progression of IgAN can be slowed by antiproteinuric therapy. Therefore, targeting proteinuria is a valid surrogate for individualized renoprotective therapy [2–4].

Hydroxychloroquine (HCQ), an immunomodulator widely used to treat autoimmune and inflammatory diseases [5–7], has recently been identified as an effective and safe antiproteinuric agent for treating IgAN [8]. A prospective study by Liu et al. found that HCQ, in addition to optimized renin-angiotensin-­aldosterone system inhibitor therapy, significantly reduced proteinuria in patients with IgAN over 6 months [9]. Additionally, Yang et al. compared the efficacy and safety of HCQ and corticosteroids in IgAN patients with persistent proteinuria and found that the time-averaged proteinuria and the cumulative frequency of patients with a 50% reduction in proteinuria within 6 months of follow-up were comparable between the HCQ and corticosteroid groups. However, HCQ was safer than corticosteroids [10]. Therefore, HCQ has been recommended for Chinese patients with a high risk of IgAN progression in the 2021 edition of Kidney Disease: Improving Global Outcomes (KDIGO) [11]. HCQ has a longer half-life (40–60 days) owing to its large volume of distribution in the blood, and its ability to disperse into the aqueous cellular and intercellular compartments results in long mean residence times (approximately 1300 h for HCQ) [5]. Moreover, HCQ interferes with lysosomal activity and autophagy, influences membrane stability, and alters signaling pathways and transcriptional activity, which can lead to the inhibition of cytokine production and the modulation of certain co-stimulatory molecules [5]. These characteristics of action, together with the chemical properties of the drug, raise the question of the optimal duration for evaluating its therapeutic efficacy in IgAN patients. From a pharmacokinetic perspective, Tett et al. showed that the blood concentrations of HCQ and its metabolites tend to stabilize after continuous use for a minimum of 6 months [12]. Therefore, according to current literature, clinical studies using HCQ to treat IgAN usually have a six-month study period to assess its effect on proteinuria [9,10,13,14]. However, according to the KDIGO guidelines, patients with IgAN should undergo an efficacy evaluation after 3 months of treatment. This is because a 24-h urine protein exceeding 0.75 g per day for 3 months is a potential risk factor for chronic kidney disease (CKD) [11]. Therefore, in addition to monitoring proteinuria, it is unclear whether other clinical indicators can predict the efficacy of IgAN treatment.

CKD is associated with substantial changes in lipid and lipoprotein metabolism, which can potentially contribute to impaired kidney function [15]. HCQ exerts beneficial effects on lipid levels. Morris et al. demonstrated that HCQ use in a rheumatoid arthritis (RA) cohort was independently associated with a significant decrease in low-density lipoprotein cholesterol (LDL-C), total cholesterol (TC), LDL-C/high-density lipoprotein cholesterol (HDL-C), and TC/HDL-C ratio. Although HCQ has only modest disease-modifying effects in the treatment of RA, its positive effects on lipid profiles make it a beneficial first-line or adjunct therapy for RA patients, particularly those with conventional cardiovascular disease risk factors [16]. Abdalla et al. reported that HCQ use in addition to low-dose steroids may reduce the lipid profile of systemic lupus erythematosus (SLE) patients and control hyperlipidemia, which can favorably affect renal disease associated with SLE [17]. Therefore, controlling the lipid levels can reduce renal damage to some degree in patients with SLE. The lipid ratio is a more accurate predictor of kidney disease risk than individual lipid components [18]. The TC/HDL-C ratio is significantly associated with an elevated risk of renal insufficiency in men, highlighting it as an independent risk factor for CKD progression [19,20]. Additionally, lipid levels considerably affect the prognosis of patients with IgAN [21]; however, it is unclear whether HCQ can reduce lipid levels in patients with IgAN and early reduction of lipids, especially the TC/HDL-C ratio, can predict the effectiveness of HCQ in IgAN. The present study was conducted to address these questions.

## Methods

### Patients

**Figure 1.**
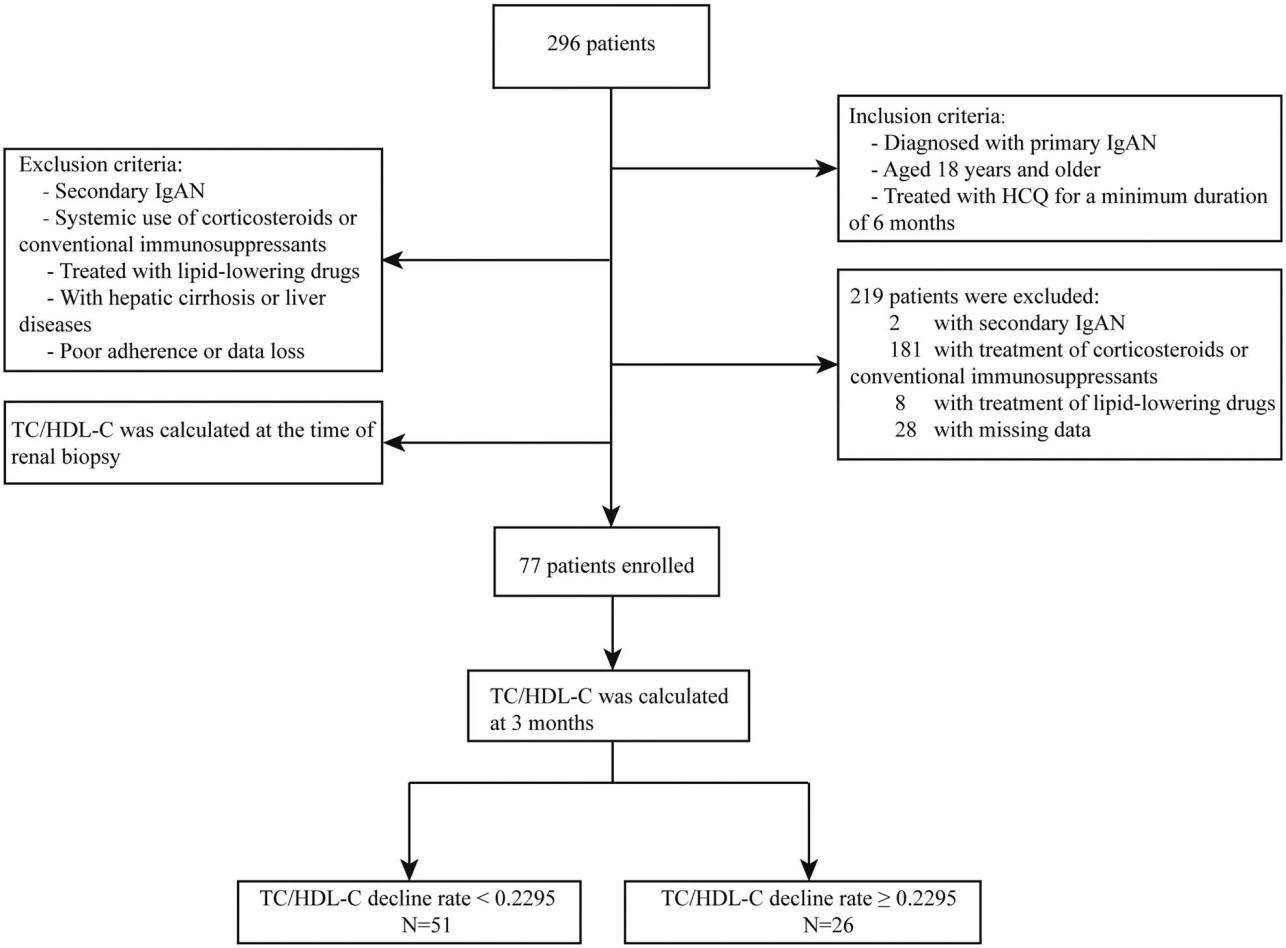
Trial profile. IgAN: immunoglobulin A nephropathy; TC: total cholesterol; HDL-C: high-density lipoprotein cholesterol.

This study enrolled 296 patients with a pathological diagnosis of IgAN at Nanjing Drum Tower Hospital between 2017 and 2023. The principal inclusion criteria were patients aged ≥18 years with biopsy-confirmed primary IgAN who had been treated with HCQ for at least 6 months. The primary exclusion criteria were as follows: secondary IgAN, systemic treatment with corticosteroids or immunosuppressants, treatment with lipid-lowering drugs, patients with cirrhosis or liver disease, and those with poor compliance or missing data. Finally, 77 adult patients (age >18 years) with biopsy-confirmed primary IgAN were treated with HCQ. A flowchart of this process is shown in Figure 1. The study was conducted in compliance with the Declaration of Helsinki and approved by the ethics committee of the Nanjing Drum Tower Hospital (approval no. 2024-196-01). Informed consent was obtained from each patient or their legal guardian prior to treatment.

### Clinical data

Baseline data, including patient demographics and laboratory parameters, were collected at the time of renal biopsy. Following the confirmation of the pathological diagnosis, HCQ therapy was typically initiated within 1 week of renal biopsy. Patient data were collected during outpatient follow-up visits scheduled every 1–3 months. In addition, patients were required to visit an ophthalmologist for eye examinations every 6 months after receiving HCQ treatment. Patient information, including age, sex, mean arterial pressure (MAP), body mass index (BMI), clinical manifestations, laboratory indices, renal pathology reports, treatment strategies, medication compliance, and adverse events, was obtained from electronic medical records. The laboratory values measured in this study included 24-h proteinuria (UPRO), serum albumin (ALB), serum creatinine (SCr), estimated ­glomerular filtration rate (eGFR), uric acid (UA), blood urea nitrogen (BUN), triglycerides (TG), TC, HDL-C, and LDL-C. The TC/HDL-C ratio was calculated by dividing serum TC by HDL-C, and the TG/HDL-C ratio was obtained by dividing serum TG by HDL-C. A 3-month time point was chosen based on previous studies to observe the effect of HCQ on lipid metabolism and to compare changes in lipid levels between different patient groups, as this may help predict long-term outcomes [22]. The eGFR was calculated using the CKD Epidemiology Collaboration creatinine equation [23]. Renal biopsy samples were evaluated by an experienced pathologist and nephrologist according to the Oxford classification [24].

### Treatments

All patients received HCQ and optimal supportive treatment, including a full dose of angiotensin-converting enzyme inhibitors or angiotensin receptor blockers. The HCQ dose varied according to the baseline eGFR values. Patients with an eGFR >60 mL/min/1.73 m^2^ and those with an eGFR of 30–60 mL/min/1.73 m^2^ received HCQ doses of 0.2 g twice daily and 0.1 g two or three times daily, respectively. However, patients with an eGFR of 15–30 mL/min/1.73 m^2^ received 0.1 g once daily.

### Outcome definition and follow‑up

A favorable outcome was defined as a reduction in proteinuria to at least 50% of the baseline values. Patients were observed for a minimum of 6 months after the initiation of HCQ treatment until either a favorable outcome was achieved or HCQ treatment was discontinued. The follow-up process is shown in the timeline infographics (Figure S1).

### Statistical analysis

Continuous variables are expressed as means ± standard deviation (SD) or medians with interquartile ranges. Categorical variables are expressed as numbers and percentages (%). Statistical analysis was performed using the Student’s *t*-test or the Mann–Whitney *U* test for continuous variables and the *χ*^2^ test for categorical variables. The optimal thresholds for the decline rate of the TC/HDL ratio were determined using receiver operating characteristic (ROC) curve analysis based on the highest Youden’s index. The Kaplan–Meier curve was used to estimate the probability of UPRO declining to less than 50% of baseline in each group. Univariate and multivariate Cox proportional hazards models were used to evaluate the therapeutic efficacy of IgAN treatment. ROC curves were used to assess the predictive values of the HCQ and metabolite blood concentrations. Data were analyzed using SPSS 26.0 software (SPSS, Chicago, IL, USA) and GraphPad Prism 9.0 (La Jolla, CA, USA). A two-tailed *p*-value of less than 0.05 was considered statistically significant.

## Results

### Demographic and clinicopathological characteristics

**Figure 2.**
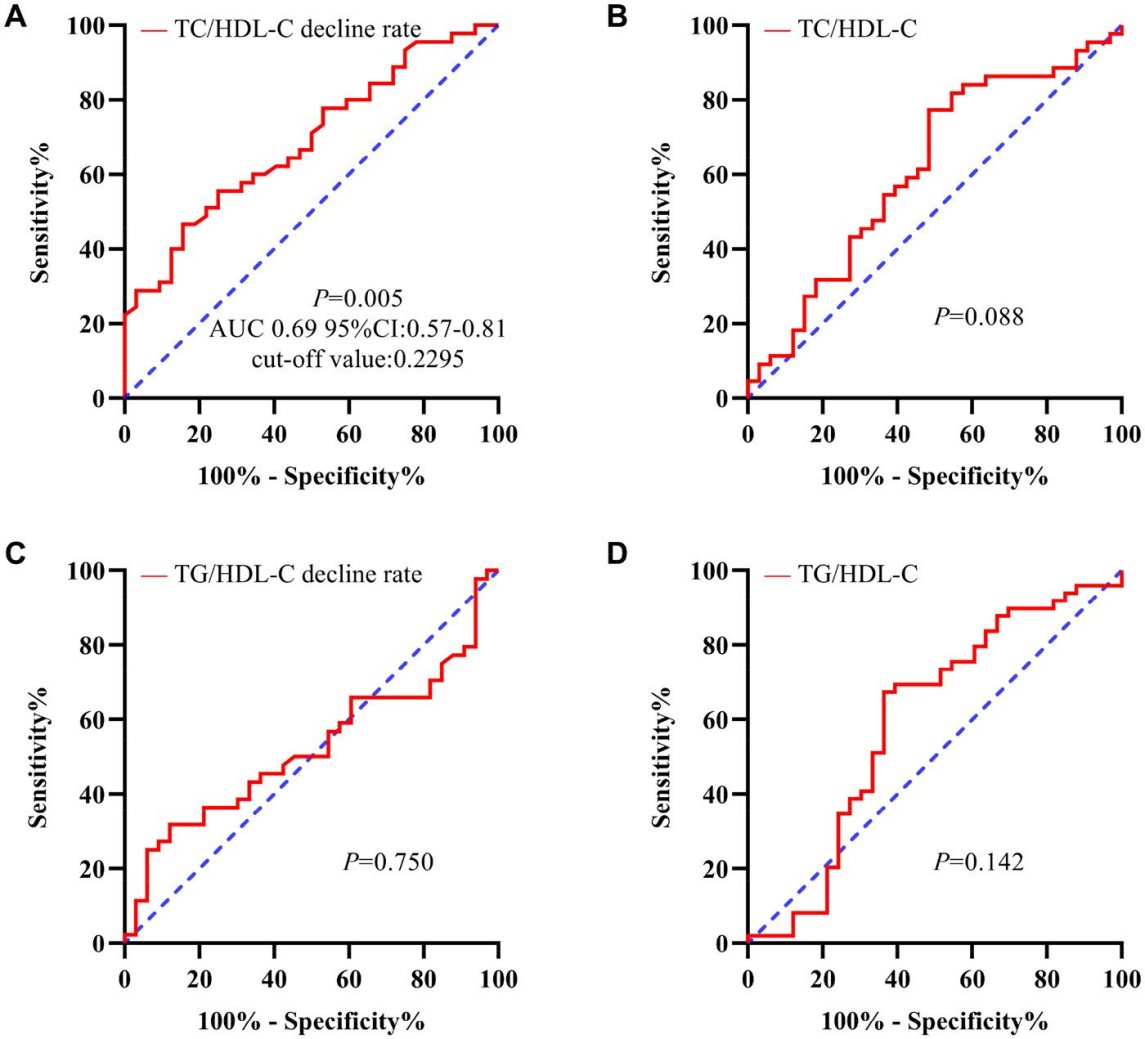
Receiver operating characteristic (ROC) curves of the TC/HDL-C decline rate. (A) TC/HDL-C, (B) TG/HDL-C decline rate, (C) and TG/HDL-C, (D) in distinguishing the therapeutic efficacy of HCQ in IgAN. IgAN: immunoglobulin A nephropathy; TC: total cholesterol; TG: triglyceride; HDL-C: high-density lipoprotein cholesterol.

This retrospective study included 77 patients with biopsy-confirmed IgAN from the Nanjing Drum Tower Hospital (Figure 1). Figure 2 shows the ROC curve comparing the predictive ability of the four variables (TC/HDL-C ratio, TG/HDL-C ratio, rate of decline in TC/HDL ratio, and TG/HDL ratio at 3 months) for the therapeutic efficacy of HCQ in the study population. The ROC curve indicated that only the rate of decline in the TC/HDL ratio at 3 months could differentiate between patients with good and poor outcomes, as the area under the curve (AUC) was statistically significant (*p* = 0.005). Moreover, ROC analysis showed that a TC/HDL ratio decline rate of 0.2295 was the optimal cutoff for predicting the therapeutic efficacy of HCQ in patients with IgAN (Figure 2). Therefore, based on the rate of decline in TC/HDL ratio at 3 months, patients were divided into two groups: high TC/HDL-C decline rate group (TC/HDL ≥0.2295, *N* = 26) and low TC/HDL-C decline rate group (TC/HDL <0.2295, *N* = 51). Baseline demographic characteristics, clinical indices including UPRO and Scr, and pathological characteristics were not significantly different between the two groups. During follow-up, the proportion of patients in the high TC/HDL-C decline rate group with a decreased proteinuria rate exceeding 50% was significantly higher than that in the low TC/HDL-C decline rate group (*p* = 0.005) (Table 1).

**Table 1.** Demographic and clinicopathological characteristics of 77 IgAN patients.

Parameters	Total (*N* = 77)	TC/HDL-C decline rate < 0.2295 (*N* = 51)	TC/HDL-C decline rate ≥ 0.2295 (*N* = 26)	*p*
Age (year)	33 (26–45)	35 (29–46)	27 (25–38)	0.037
Sex (male, %)	38 (49.3)	25 (49)	13 (50)	0.935
MAP (mmHg)	103.0 ± 13.2	102.5 ± 14	103.8 ± 11.6	0.683
BMI (kg/m2)	23.9 (21.7–26)	24.2(22–26)	23.4 (20.9–25.9)	0.368
Hypertension (%)	5 (6.5)	2 (3.9)	3 (11.5)	0.329
CKD stages (%)				
Stage 1	47 (61)	34 (66.6)	13 (50)	0.156
Stage 2	24 (31.1)	14 (27.4)	10 (38.4)	0.324
Stage 3	6 (7.7)	3 (5.8)	3 (11.5)	0.381
Pathologic				
M1 (%)	77 (100)	51 (100)	26 (100)	1.000
E1 (%)	0 (100)	0 (0)	0 (0)	1.000
S1 (%)	22 (28.5)	37 (72.5)	18 (69.2)	0.762
T1-2/T0 (%)	11 (14.2)	5 (9.8)	6 (23)	0.118
C1-2/C0 (%)	34 (44.1)	22 (43.1)	12 (46.1)	0.802
Clinical				
Scr (umol/L)	75 (58–95.5)	74 (58–89)	81 (57.8–108.3)	0.176
eGFR (ml/min/1.73 m2)	100.6 (80.3–120)	102.8 (85.6–118.2)	89.6 (74.9–122.7)	0.575
ALB (g/L)	40.2 ± 3.2	40.5 ± 3.1	39.6 ± 3.4	0.237
HDL-C (mmol/L)	1.09 (0.94–1.43)	1.19 (1.00–1.43)	1.00 (0.79–1.42)	0.14
LDL-C (mmol/L)	2.74 ± 0.73	2.65 ± 0.70	2.92 ± 0.78	0.124
TG (mmol/L)	1.34 (0.98–1.90)	1.42 (0.94–1.97)	1.24 (1.00,1.89)	0.674
TC (mmol/L)	4.62 ± 0.90	4.54 ± 0.80	4.77 ± 1.08	0.297
UPRO (mg/d)	965 (617.5–1518.5)	921 (597–1505)	1036 (648–2062)	0.343
BUN (mmol/L)	5.1 (4.2–6.1)	5.1 (4.4–6.0)	5.1 (3.8–6.2)	0.800
UA (umol/L)	359.4 ± 97.9	357.8 ± 93.6	362.6 ± 107.5	0.839
Follow-up				
Duration (months)	6.0 (2.0–12)	6.0 (3.0–12)	3.5 (2.0–6.0)	
Patients reached the endpoint at 6 months (%)	41 (53.2)	22 (43.1)	19 (73.1)	
UPRO decline rate ≥50% (%)	45 (58.4)	24 (47.1)	21 (80.8)	0.005

MAP: mean arterial pressure; BMI: body mass index; CKD: chronic kidney disease; M: mesangial proliferation; E: endocapillary proliferation; S: segmental sclerosis; T: tubular atrophy/interstitial fibrosis; C: crescents; Scr: serum creatinine; eGFR: estimated glomerular filtration rate; ALB: albumin; HDL-C: high-density lipoprotein cholesterol; LDL-C: low-density lipoprotein cholesterol; TG: triglycerides; TC: total cholesterol; UPRO: 24 h urine protein; BUN: blood urea nitrogen; UA: uric acid.

### Correlation of the TC/HDL-C ratio with clinical variables

To investigate the relationship between the TC/HDL-C ratio and other clinical indicators, correlation analysis was performed, which revealed a significant negative correlation between the TC/HDL-C ratio and eGFR (*r* = −0.411, *p* < 0.001). In addition, the TC/HDL-C ratio was positively correlated with UPRO (*r* = 0.242, *p* = 0.034), BMI (*r* = 0.326, *p* = 0.004), and BUN (*r* = 0.335, *p* = 0.003) (Table 2).

**Table 2.** Correlation between related variables and TC/HDL-C.

	Variables	Correlation coefficient (*r*)	*p* Value
TC/HDL-C	UPRO	0.242	0.034
	UA	0.193	0.094
	BMI	0.326	0.004
	eGFR	−0.411	<0.001
	ALB	−0.018	0.874
	BUN	0.335	0.003

TC: total cholesterol; HDL-C: high-density lipoprotein cholesterol; UPRO: 24-h urine protein; UA: uric acid; BMI: body mass index; eGFR: estimated glomerular filtration rate; ALB: albumin; BUN: blood urea nitrogen.

### Changes in clinical variables with treatment of HCQ

**Figure 3.**
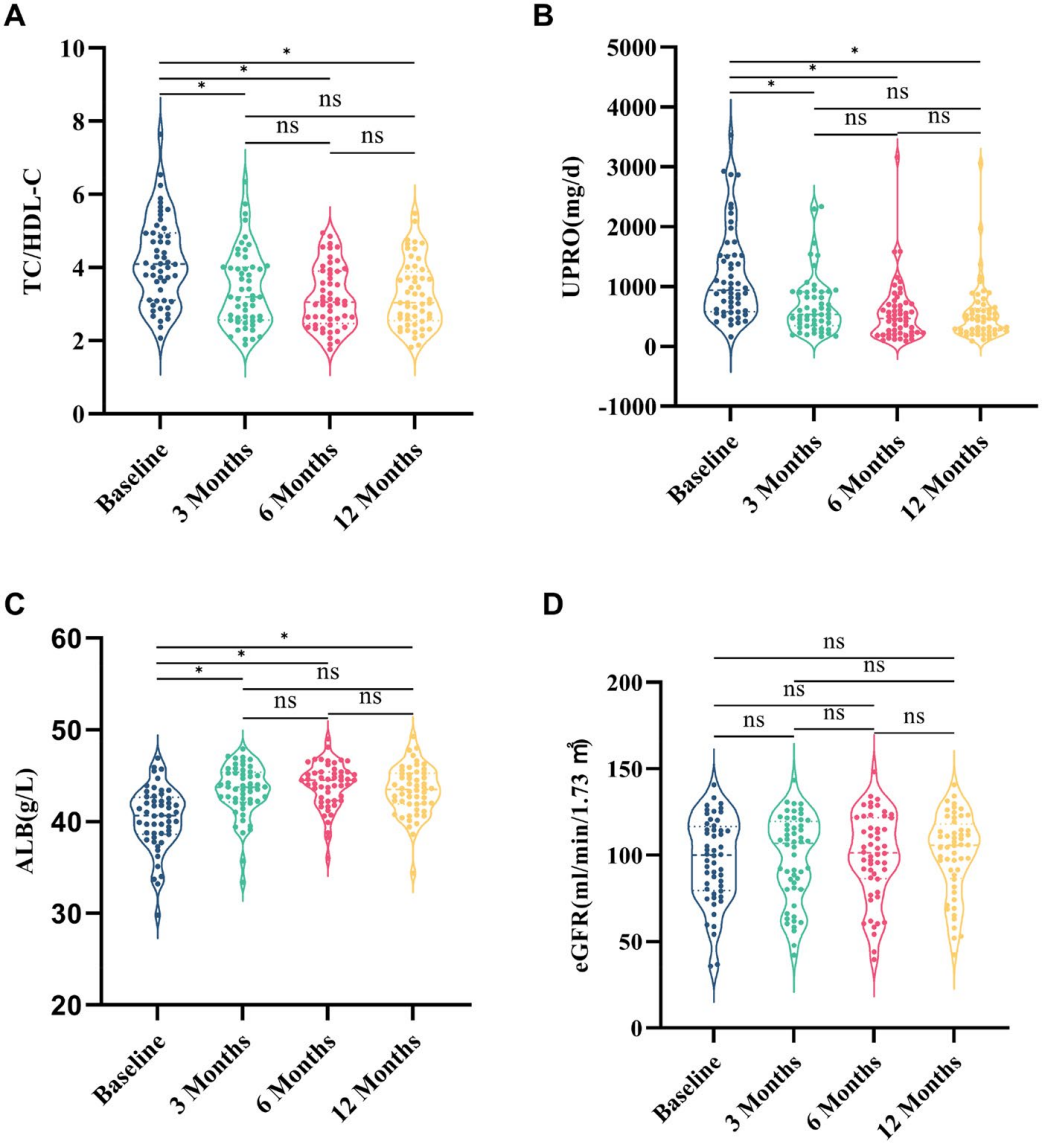
Changes in clinical variables from baseline to 12 months of follow-up TC/HDL-C (A), UPRO (B), ALB (C), and eGFR (D). *p < 0.001. TC: total cholesterol; HDL-C: high-density lipoprotein cholesterol; UPRO: 24-hour urine protein; ALB: albumin; eGFR: estimated glomerular filtration rate.

The results illustrating the impact of HCQ on clinical indicators in patients with IgAN are shown in Figure 3. Our findings indicated that HCQ treatment significantly decreased TC/HDL-C ratio (4.10 [IQR, 3.10, 4.94] g/day vs. 3.20 [IQR, 2.56, 4.01]; *p* < 0.001) and UPRO levels (943 [IQR, 583, 1522] mg/day vs. 538 [IQR, 348, 913] mg/day; *p* < 0.001) in patients after 3 and up to 12 months, respectively. However, no significant changes in these indicators were observed between 3 and 12 months. Additionally, the administration of HCQ resulted in a notable increase in ALB levels (40.3 ± 3.4 g/L vs. 43.4 ± 2.8 g/L; *p* < 0.001) inpatients after 3 months of treatment. However, the eGFR levels of patients remained stable throughout the entire 12 months of treatment (Figure 3).

**Figure 4.**
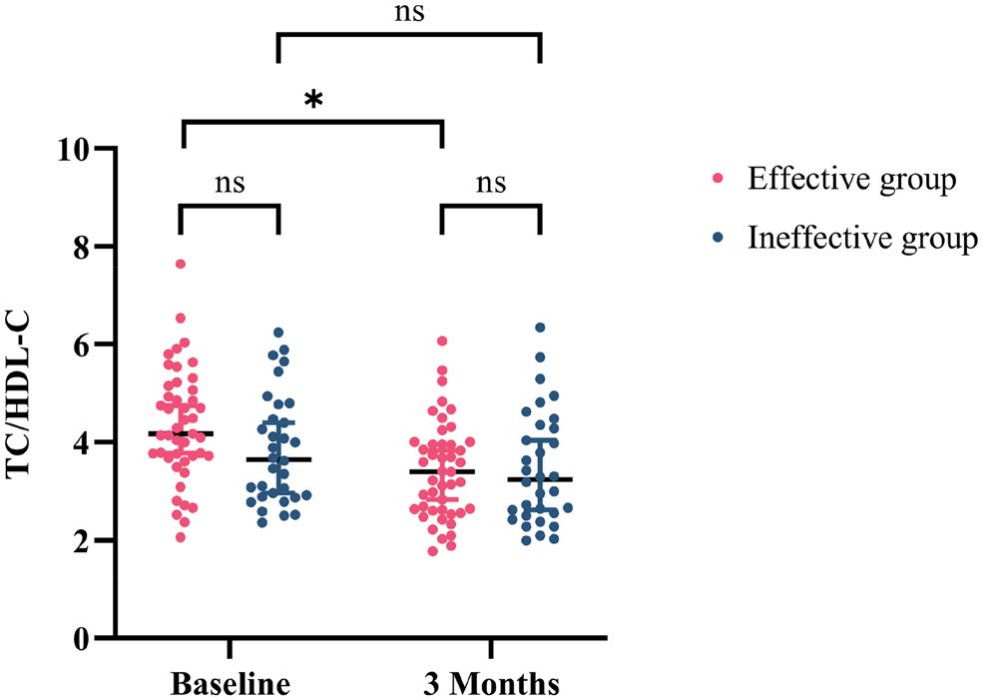
Comparison of the changes in TC/HDL-C at baseline and after 3 months of treatment between the treatment-effective and treatment-ineffective groups (*p < 0.001).

Based on the treatment outcomes, patients were divided into two groups: treatment-effective (*n* = 45) and treatment-ineffective (*n* = 32) groups. The TC/HDL-C levels at baseline and 3 months after treatment were compared between the two groups (Figure 4). The results showed that lipid levels were not significantly different at baseline (4.177 [IQR, 3.710, 5.113] vs. 3.657 [IQR, 2.903, 4.705]; *p* = 0.064) and 3 months after treatment (3.408 [IQR, 2.621, 3.986] vs. 3.246 [IQR, 2.527, 4.347]; *p* = 0.852). However, compared with baseline, patients in the treatment-effective group demonstrated a significant improvement in TC/HDL-C levels after 3 months of treatment (4.177 [IQR, 3.710, 5.113] vs. 3.408 [IQR, 2.621, 3.986]; *p* < 0.001), whereas no significant changes were observed in patients in the treatment-ineffective group (3.657 [IQR, 2.903, 4.705] vs. 3.246 [IQR, 2.527, 4.347]; *p* = 0.170).

### Rate of decline in TC/HDL-C ratio as an independent predictor of better efficacy of HCQ for IgAN treatment

**Figure 5.**
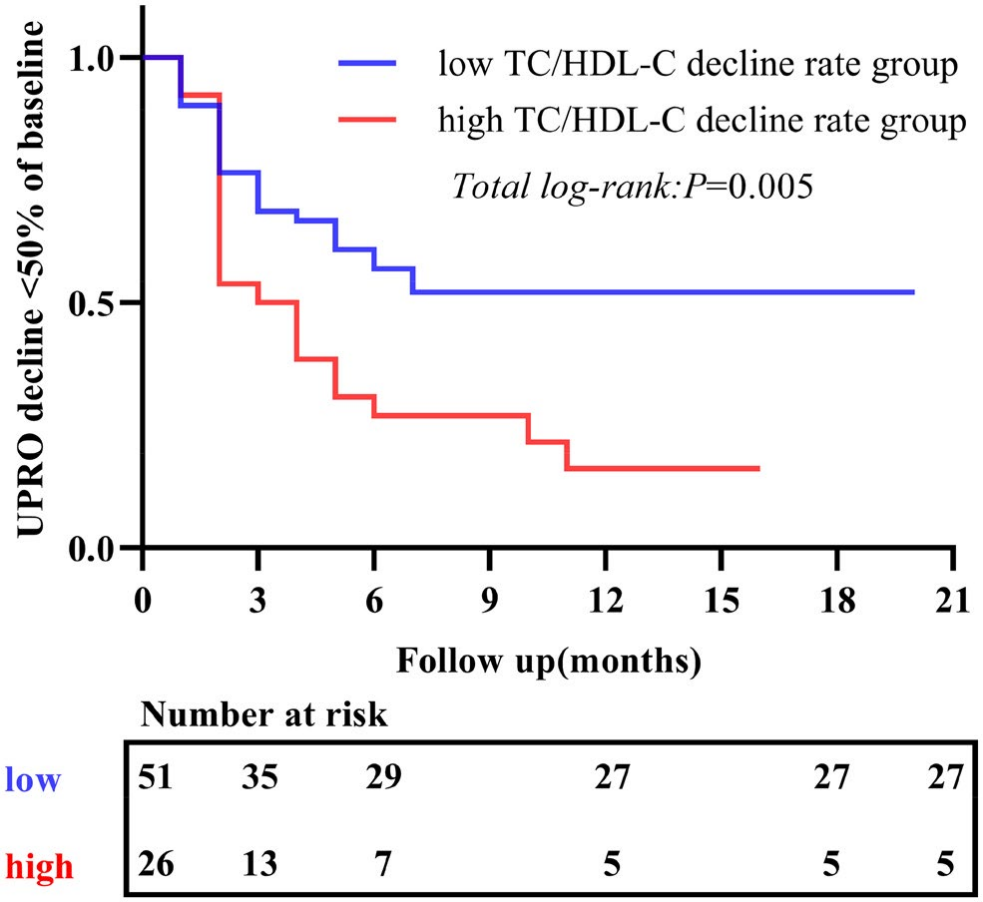
Kaplan–Meier survival for UPRO decline was less than 50% of baseline in patients with low or high TC/HDL-C decline rates. TC: total cholesterol; HDL-C: high-density lipoprotein cholesterol; UPRO: 24-hour urine protein.

A total of 45 (58.4%) patients exhibited *a* ≥ 50% decline in UPRO throughout the follow-up period. Kaplan–Meier analysis demonstrated a significant correlation between *a* ≥ 0.2295 decline in TC/HDL-C at 3 months and *a* ≥ 50% decline in UPRO (Figure 5). Furthermore, Cox regression analysis identified factors contributing to *a* ≥ 50% decline in UPRO in IgAN patients. Before using the Cox regression model, a series of statistical tests and graphical diagnostics were conducted to determine the validity of the proportional risk assumption. Specifically, the Schoenfeld residual test was used for continuous variables, and the Schoenfeld residuals were plotted for each covariate (Supplementary Figure S2). The diagnostic results indicated that the proportional risk assumption for the included variables was valid (*p* > 0.05) (Supplementary Table S1). For categorical variables, the proportionality hypothesis was tested by plotting log-minus-log survival curves (Supplementary Figure S3). The results suggested that the proportional risk hypothesis was valid. Univariate analysis demonstrated that a higher rate of decline in the TC/HDL-C ratio was associated with a more favorable prognosis in patients treated with HCQ (hazard ratio [HR] = 2.049, 95% CI: 1.125–3.734, *p* = 0.019). To exclude the effect of relevant confounders, variables associated with TC/HDL-C (Table 2) and those with a *p* value ˂ 0.1 in the univariate Cox regression analysis were included in the multivariate Cox regression analysis. The results demonstrated that a higher rate of decline in the TC/HDL-C ratio (HR 2.314, 95% CI 1.234–4.340; *p* = 0.009) was a more accurate predictor of ≥ 50% decline in proteinuria in IgAN patients treated with HCQ (Table 3).

**Table 3.** Analysis of factors associated with HCQ efficacy in the treatment of IgAN.

		Univariate			Multivariate	
Parameter	HR	95%CI	*p* value	HR	95%CI	*p* value
High TC/HDL-C decline rate	2.049	1.125–3.734	0.019	2.314	1.234–4.340	0.009
Male	1.785	0.976–3.265	0.060	1.668	0.830–3.355	0.151
Age (per year)	0.979	0.953–1.006	0.133	–	–	–
BMI (kg/m^2^)	1.003	0.923–1.089	0.943	0.963	0.879–1.054	0.411
MAP (mmHg)	1.002	0.979–1.025	0.887	–	–	–
S1	0.915	0.471–1.777	0.793	–	–	–
T_1-2_/T_0_	1.489	0.662–3.350	0.336	–	–	–
C_1-2_/C_0_	1.152	0.637–2.083	0.639	–	–	–
CKD stages	0.988	0.631–1.545	0.956	–	–	–
Scr (umol/L)	1.005	0.995–1.015	0.345	–	–	–
eGFR	0.998	0.987–1.010	0.770	0.994	0.976–1.013	0.542
ALB (g/L)	1.098	0.993–1.215	0.068	1.115	0.997–1.247	0.057
BUN (mmol/L)	0.983	0.811–1.191	0.859	0.889	0.666–1.185	0.422
UA (umol/L)	1.002	0.999–1.005	0.105	–	–	–
TC (mmol/L)	1.050	0.744–1.484	0.780	–	–	–
TG (mmol/L)	1.006	0.710–1.424	0.975	–	–	–
LDL (mmol/L)	1.244	0.818–1.892	0.307	–	–	–
HDL (mmol/L)	0.507	0.204–1.259	0.144	–	–	–

BMI: body mass index; MAP: mean arterial pressure; S: segmental sclerosis; T: tubular atrophy/interstitial fibrosis; C: crescents; CKD: chronic kidney disease; Scr: serum creatinine; eGFR: estimated glomerular filtration rate; ALB: albumin; BUN: blood urea nitrogen; UA: uric acid; TC: total cholesterol; TG: triglycerides; LDL-C: low-density lipoprotein cholesterol; HDL-C: high-density lipoprotein cholesterol.

### Safety and adverse events

Gastrointestinal symptoms were reported in four patients, and corticosteroid or other immunosuppressive therapies were initiated within the first 3 months of HCQ treatment. No instances of hospitalization or mortality due to adverse events were reported.

## Discussion

Patients with IgAN typically experience chronic, slowly progressive kidney injury. Lower levels of proteinuria are often associated with a slower decline in renal function and a reduced risk of CKD [2–4]. HCQ demonstrated a safe and reliable anti-proteinuric effect in patients with IgAN. In addition to reducing proteinuria, HCQ improves lipid levels in patients with autoimmune disorders [16,17]. Moreover, abnormal lipid metabolism is an independent risk factor for IgAN progression to end-stage renal disease [21]. However, no studies have examined the effect of HCQ on lipid metabolism in patients with IgAN, and it is unclear whether improving lipid levels affects the efficacy of treatments such as reducing proteinuria.

Lipid ratios are more accurate predictors of disease activity than individual lipid components [25,26]. In recent years, lipid ratios such as TC/HDL-C and TG/HDL-C have been proposed as alternative biomarkers for predicting Type 2 diabetes mellitus, cardiovascular disease, and metabolic syndrome [26,27]. Furthermore, TC/HDL-C is a potentially valuable biomarker for assessing the severity of alveolar proteinosis [28]. Additionally, Schaeffner et al. observed a positive association between high TC/HDL-C ratios and an increased risk of renal failure in apparently healthy men [19]. This present study was the first to evaluate the association between changes in TC/HDL-C ratio and HCQ efficacy in patients with IgAN. ROC curves were employed to assess the ability of the TC/HDL-C and TG/HDL-C ratios and the early rate of decline of both ratios during HCQ therapy to predict *a* ≥ 50% reduction in proteinuria. The rate of decline in the TC/HDL-C ratio was the only statistically significant predictor of HCQ efficacy.

Liu et al. and Yang et al. conducted prospective and retrospective clinical studies, respectively, on the therapeutic effects of HCQ in patients with IgAN over a six-month period. Both studies found that HCQ effectively decreased proteinuria and maintained stable eGFR levels in these patients [9,10]. Our long-term observational study demonstrated that HCQ treatment for IgAN was effective in reducing proteinuria and increasing ALB levels in patients at 3 months, and these effects were sustained for up to 12 months. However, eGFR levels did not change significantly throughout the treatment course. Our findings were consistent with those of previous studies and provided further evidence on the efficacy and safety of HCQ in the treatment of IgAN. Furthermore, we examined the impact of HCQ on the lipid profiles of patients with IgAN and explored the association between alterations in lipid profiles and a reduction in proteinuria. Our findings revealed that HCQ markedly improved TC/HDL-C levels in patients with IgAN during the early stages of treatment, particularly at 3 months, which corroborated the findings of Cairoli et al. in the SLE population [22]. At the cellular level, chloroquine inhibits cholesterol synthesis by preventing the lysosomal hydrolysis of cholesteryl esters. Moreover, HCQ enhanced the activity of 3-hydroxy-3-methyl-gutaryl-CoA reductase and slowed down the degradation of the enzyme, resulting in decreased lipid levels [29,30]. This may explain the mechanism by which HCQ improves lipid levels in patients with IgAN. Patients were further grouped according to the TC/HDL-C decline rate. Kaplan–Meier curves revealed that patients in the high decline rate group were more likely to achieve favorable outcomes. Univariate Cox regression analysis demonstrated that a high TC/HDL-C decline rate was significantly associated with improved outcomes (HR = 2.049, 95% CI: 1.125–3.734, *p* = 0.019). After adjusting for potential confounding variables, multivariate Cox regression analysis revealed that a high TC/HDL-C decline rate remained a significant predictor of improved outcomes (HR = 2.314, 95% CI: 1.234–4.340, *p* = 0.009).

Lipid metabolism disorders are closely associated with the outcome and prognosis of renal diseases. Moorhead et al. demonstrated that the loss of proteinuria leads to compensatory reductions in the synthesis and catabolism of lipoproteins in the liver, which in turn leads to dyslipidemia and additional renal damage [31]. Lu et al. demonstrated that acetyl-CoA synthetase 2, a core metabolic intermediate in transcriptional regulation and a substrate for *de novo* lipogenesis, can cause podocyte damage, leading to albuminuria and deterioration of diabetic renal function [32]. The results showed a positive correlation between TC/HDL-C levels and UPRO (*r* = 0.242, *p* = 0.034) and a negative correlation between TC/HDL-C levels and eGFR levels (*r* = −0.411, *p* < 0.001) in patients with IgAN. Furthermore, the patients were grouped according to the efficacy of HCQ treatment, and their lipid levels were compared at baseline and after treatment. Despite no significant difference in lipid levels at baseline, patients with a good response to HCQ treatment demonstrated a significant decrease in TC/HDL-C levels, whereas patients with a poor response showed no significant improvement in lipid levels. Consequently, there may be a correlation between the ability of HCQ to reduce proteinuria and its lipid-lowering efficacy. This correlation may permit the use of HCQ as a predictor of the ability to reduce proteinuria by examining the extent of early TC/HDL-C improvement.

Notably, lipids are closely associated with the pathological damage in IgAN. Zhuang et al. demonstrated that children with dyslipidemia exhibited more severe clinical features and pathological changes and a higher proportion of S1 and C2 in the Oxford classification of IgAN than those of children without dyslipidemia [33]. Choi et al. revealed that the overall degree of nephrosclerosis (S) was higher in patients with IgAN in the hyperlipidemic group than in the normolipidemic group [34]. This may be attributed to the fact that dyslipidemia can result in tubular injury and promote renal fibrosis by inducing lipotoxicity, inflammation, oxidative stress, and signal transduction events [35]. In our study, the group with a low rate of TC/HDL-C decline also exhibited a higher S1 score than the group with a high rate of decline (72.5 vs. 69.2%), although the difference between the two groups was not statistically significant. However, univariate Cox regression analysis revealed that S1 was not correlated with HCQ efficacy. This may be because of the relatively small sample size, indicating that a larger sample size is required to investigate the relationship between pathological damage and efficacy.

During the observation period, the adverse events associated with HCQ were relatively limited. Only four patients experienced adverse drug reactions at the beginning of HCQ treatment, mainly gastrointestinal reactions, including nausea (*n* = 3) and diarrhea (*n* = 1). These symptoms were alleviated following the implementation of an adjusted treatment regimen. Most importantly, no serious adverse events such as severe infections, cardiovascular events, or deaths occurred during the follow-up period. A systematic review of several major clinical studies on HCQ treatment for IgAN revealed that the most common adverse events were skin and mucosal reactions, followed by gastrointestinal and allergic reactions, and cardiovascular events were rare [36]. This finding is consistent with the conclusions of our study, which further confirms the safety of HCQ for the treatment of IgAN.

Our study had certain limitations, which should be considered. First, this was a retrospective, single-center study with a limited sample size, which restricted our ability to infer the accurate relationship between the early TC/HDL-C decline rate and the efficacy of HCQ. Therefore, these results should be interpreted with caution. Further prospective studies with extended observation times, larger sample sizes, and diverse racial and regional groups are necessary to confirm these findings. Second, our study included patients without diabetes, with a limited number of patients with hypertension, resulting in a lack of data on these populations. Third, some confounding variables, such as tobacco or alcohol use, diet, and other potential factors, were not collected, which may have affected the conclusions drawn from the data. Fourth, this study included only patients with relatively preserved eGFR, and the majority of patients exhibited mild pathological changes, which restricts the generalizability of our findings. Including a larger patient cohort with varying eGFR levels and pathological types may be beneficial. Finally, the precise mechanism by which HCQ improves IgAN lipid levels and reduces urinary protein levels requires further investigation.

## Conclusions

This study demonstrates that HCQ effectively improves the lipid profiles of patients with IgAN. Furthermore, an early decline in the TC/HDL-C ratio may serve as a predictor of HCQ efficacy in these patients.

## Supplementary Material

Supplementary Table.doc

Supplementary Figure.doc

## Data Availability

All data generated or analyzed during this study are included in this article. Further inquiries can be directed to the corresponding author.
