# Supplementary material for: Early reduction in total cholesterol to high-density lipoprotein cholesterol ratio predicts hydroxychloroquine efficacy in treating IgA nephropathy
Source: Ren Fail. 2024 Aug 30;46(2):2397046. doi: 10.1080/0886022X.2024.2397046 (PMC11370678; doi:10.1080/0886022X.2024.2397046)
Supplement: Supplementary Table.doc [file IRNF_A_2397046_SM6866.doc]

**Supplementary Table S1. Correlation analysis of variable bias residuals and rank of time.**

| **Correlations** | | | | | | | | | | | | | |
| --- | --- | --- | --- | --- | --- | --- | --- | --- | --- | --- | --- | --- | --- |
|  |  | Age | MAP | BMI | eGFR | Scr | ALB | BUN | UA | TC | TG | HDL | LDL |
| Rank of Time | Pearson Correlation | 0.163 | 0.053 | 0.205 | -0.169 | 0.113 | 0.030 | -0.002 | -0.082 | -0.075 | -0.083 | -0.143 | -0.043 |
|  | *P* value | 0.290 | 0.731 | 0.182 | 0.273 | 0.467 | 0.845 | 0.989 | 0.597 | 0.628 | 0.593 | 0.356 | 0.781 |
