# Supplementary figures and images for: Early reduction in total cholesterol to high-density lipoprotein cholesterol ratio predicts hydroxychloroquine efficacy in treating IgA nephropathy
Source: Ren Fail. 2024 Aug 30;46(2):2397046. doi: 10.1080/0886022X.2024.2397046 (PMC11370678; doi:10.1080/0886022X.2024.2397046)

**Supplementary Figure S1**


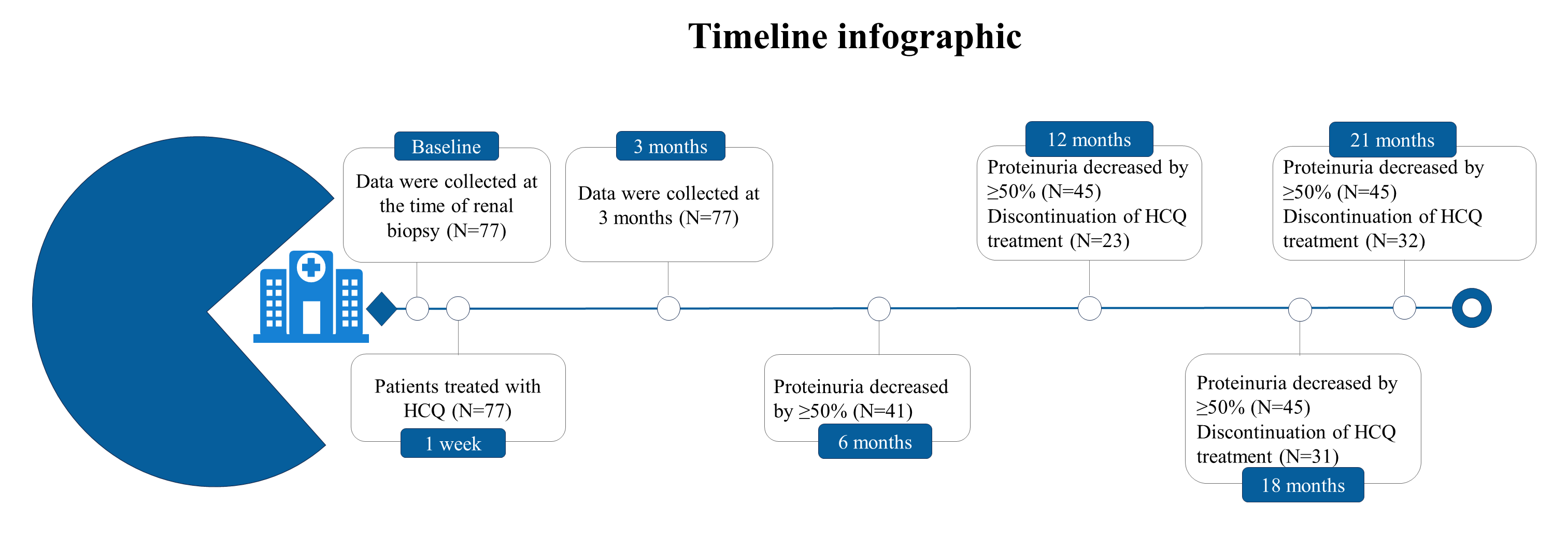


**Supplementary Figure S2**


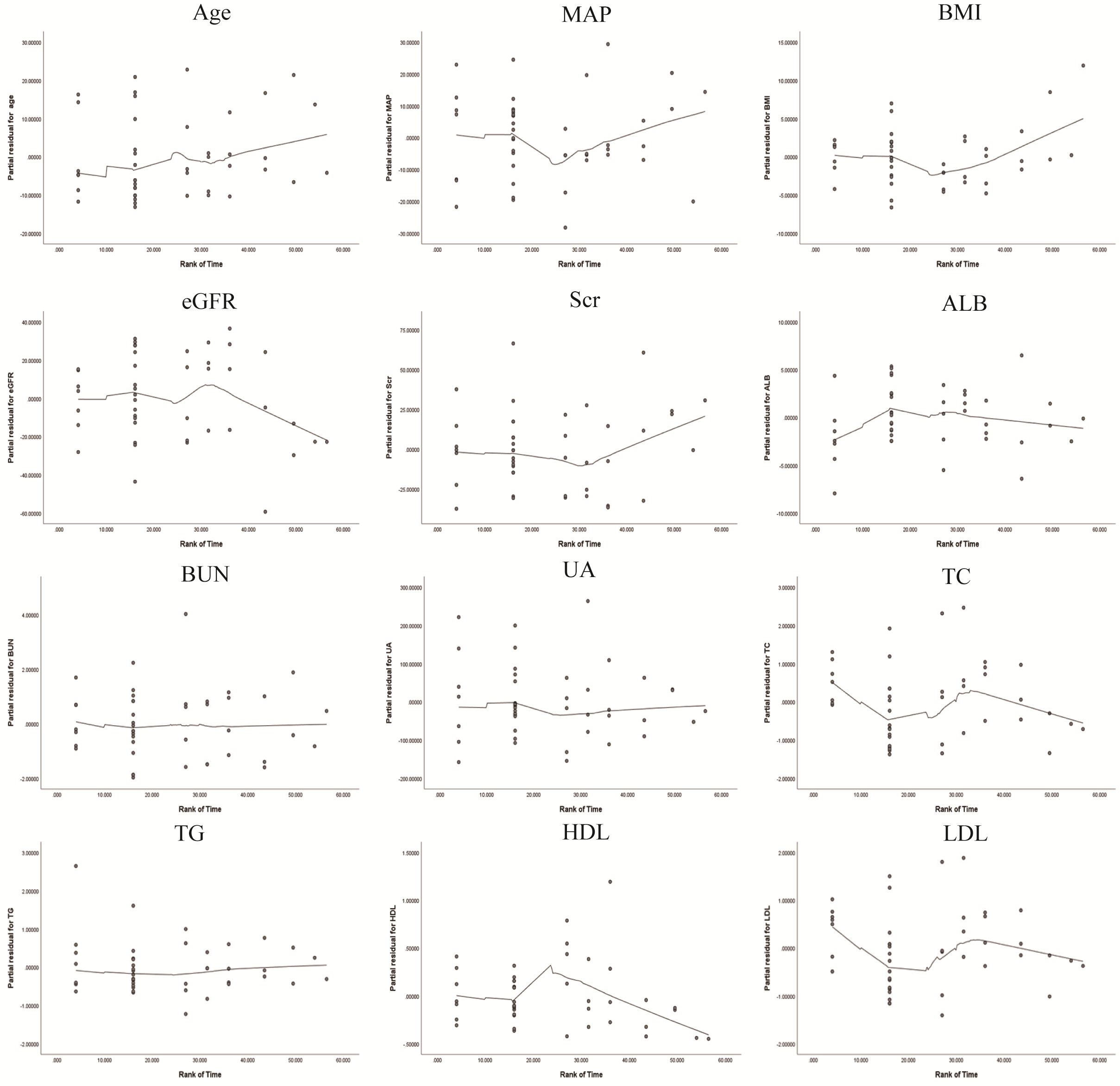


**Supplementary Figure S3**

**
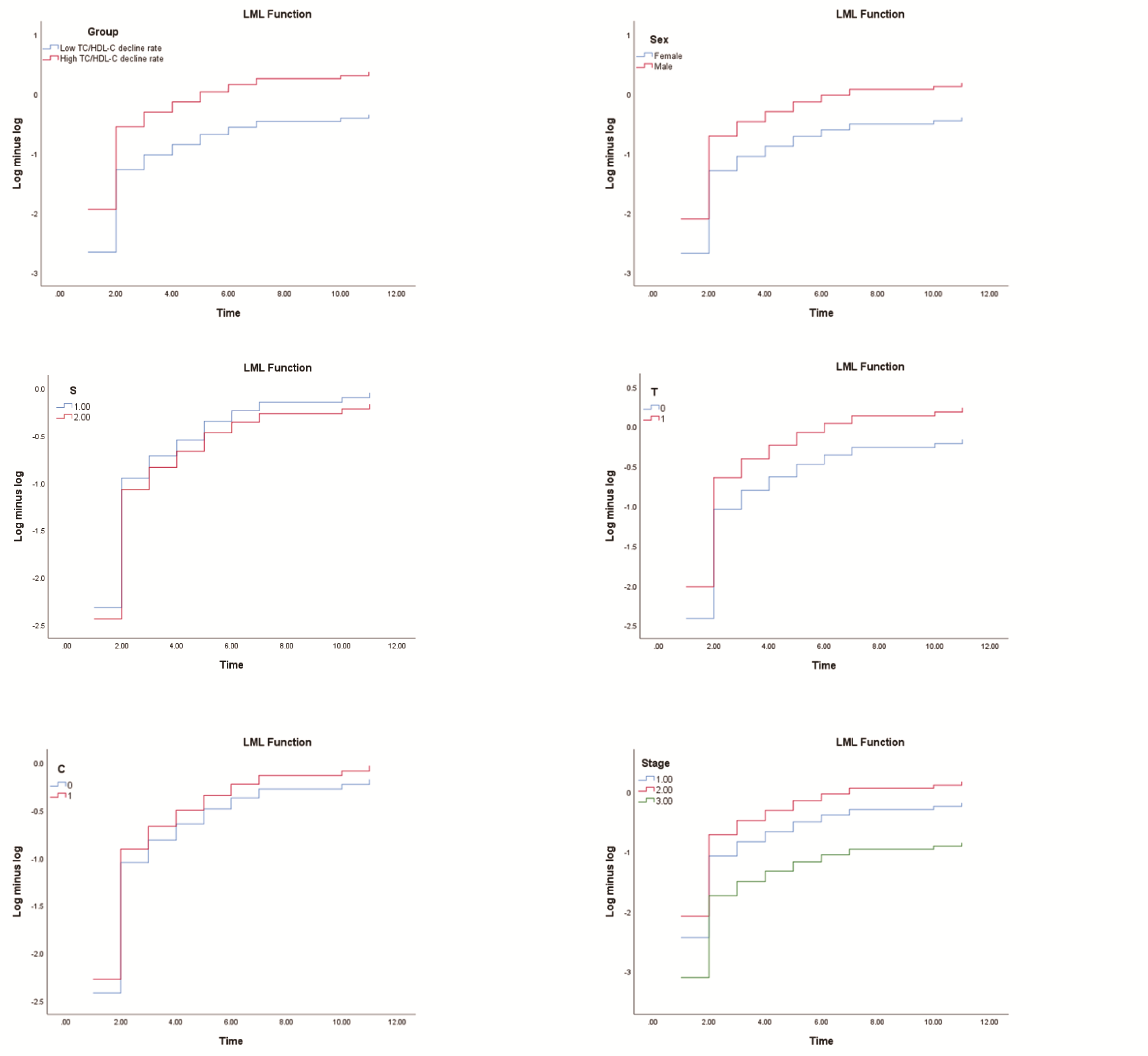
**

Supplement: Supplementary Figure.doc [file IRNF_A_2397046_SM6865.doc]
